# Immunological Signatures for Early Detection of Human Head and Neck Squamous Cell Carcinoma through RNA Transcriptome Analysis of Blood Platelets

**DOI:** 10.3390/cancers16132399

**Published:** 2024-06-29

**Authors:** Jappreet Singh Gill, Benu Bansal, Rayansh Poojary, Harpreet Singh, Fang Huang, Jett Weis, Kristian Herman, Brock Schultz, Emre Coban, Kai Guo, Ramkumar Mathur

**Affiliations:** 1 Department of Geriatrics, School of Medicine and Health Sciences, University of North Dakota, Grand Forks, ND 58202, USA; benu.bansal@und.edu (B.B.);; 2 Department of Biomedical Engineering, School of Electrical Engineering and Computer Sciences, University of North Dakota, Grand Forks, ND 58202, USA; 3 Department of Biomedical Sciences, School of Medicine and Health Sciences, University of North Dakota, Grand Forks, ND 58202, USA; 4 Department of Neurology, University of Michigan, Ann Arbor, MI 48109, USA

**Keywords:** head and neck squamous cell carcinoma, transcriptomics, precision medicine, WGCNA, network biology, tumor-educated platelet, molecular diagnostics, blood-based biomarkers, machine learning

## Abstract

**Simple Summary:**

Head and neck squamous cell carcinoma (HNSCC) remains a global health concern due to the lack of precise early diagnostic biomarkers and often-delayed diagnosis. This study employs machine learning, weighted gene co-expression network analysis, and network biology to identify transcriptomic markers for HNSCC detection. We identified nine genes with significantly differentially expressed activity in samples from HNSCC patients. These gene signatures could greatly improve early HNSCC identification and warrant further investigation to confirm their predictive and therapeutic significance. The transcriptional landscape of platelets in head and neck cancer patients revealed distinct gene expression profiles compared to healthy controls, underscoring the systemic impact of the tumor on blood platelets. Additionally, the study emphasizes the role of tumor-educated platelets (TEPs), which carry RNA signatures indicative of tumor-derived material, offering a non-invasive source for early-detection biomarkers.

**Abstract:**

Although there has been a reduction in head and neck squamous cell carcinoma occurrence, it continues to be a serious global health concern. The lack of precise early diagnostic biomarkers and postponed diagnosis in the later stages are notable constraints that contribute to poor survival rates and emphasize the need for innovative diagnostic methods. In this study, we employed machine learning alongside weighted gene co-expression network analysis (WGCNA) and network biology to investigate the gene expression patterns of blood platelets, identifying transcriptomic markers for HNSCC diagnosis. Our comprehensive examination of publicly available gene expression datasets revealed nine genes with significantly elevated expression in samples from individuals diagnosed with HNSCC. These potential diagnostic markers were further assessed using TCGA and GTEx datasets, demonstrating high accuracy in distinguishing between HNSCC and non-cancerous samples. The findings indicate that these gene signatures could revolutionize early HNSCC identification. Additionally, the study highlights the significance of tumor-educated platelets (TEPs), which carry RNA signatures indicative of tumor-derived material, offering a non-invasive source for early-detection biomarkers. Despite using platelet and tumor samples from different individuals, our results suggest that TEPs reflect the transcriptomic and epigenetic landscape of tumors. Future research should aim to directly correlate tumor and platelet samples from the same patients to further elucidate this relationship. This study underscores the potential of these biomarkers in transforming early diagnosis and personalized treatment strategies for HNSCC, advocating for further research to validate their predictive and therapeutic potential.

## 1. Introduction

Head and neck cancer (HNC), with approximately 900,000 new cases and 400,000 deaths annually worldwide, continues to pose a significant healthcare challenge [1]. The predominant form, head and neck squamous cell carcinoma (HNSCC), arises from the mucosal linings of the oral cavity, pharynx, and larynx, and despite medical advances, maintains a dismal five-year survival rate of about 60% [2,3]. This stark statistic underlines the urgent need for an improved understanding of HNSCC’s molecular underpinnings and the development of precise, targeted therapeutic strategies.

Recent genomic and epigenetic explorations have significantly advanced our knowledge of the intricate molecular landscape of HNSCC. Utilizing next-generation sequencing (NGS) and comprehensive gene expression profiling, researchers have uncovered diverse genetic and epigenetic alterations that disrupt critical oncogenic pathways, including mutations in pivotal genes such as *TP53*, *CDKN2A*, *PIK3CA*, and *HRAS* [4,5,6]. These discoveries underscore the potential of genomic insights to steer the development of novel diagnostic and therapeutic approaches.

Identifying TP53 mutations in up to 60% of HNSCC cases, which are often associated with poor outcomes and resistance to therapy, highlights the critical need for early detection strategies that could improve clinical management and patient survival [5]. Furthermore, prevalent copy number alterations, such as EGFR gene amplifications and losses in chromosome 9p, reveal potential targets for early intervention [7,8]. In addition to genomic mutations, epigenetic modifications play a crucial role in HNSCC progression. The frequent promoter hypermethylation of tumor suppressor genes suggests an avenue for early detection and intervention, potentially transforming patient prognosis [9]. Recent genomic classifications, such as The Cancer Genome Atlas (TCGA), have delineated HNSCC into subtypes with distinct molecular characteristics, offering a pathway toward personalized medicine [10,11].

Despite these advances, the heterogeneity of HNSCC, including variations across different tumor sites and between HPV-positive and HPV-negative cancers, complicates the development of universal biomarkers and targeted therapies [12,13]. This heterogeneity necessitates a more nuanced approach to biomarker discovery that embraces the disease’s complexity while seeking actionable molecular targets.

Our study leverages an integrated genomic strategy combining RNA-seq data analysis, machine learning, and network biology to identify diagnostic biomarkers for HNSCC. We have identified key gene signatures by analyzing differentially expressed genes in HNC compared to normal controls, constructing protein–protein interaction networks, and employing machine learning classifiers. These biomarkers were rigorously assessed against external datasets from TCGA and GTEx, confirming their potential to accurately distinguish HNSCC.

Additionally, this study acknowledges that platelet samples and tumor samples were derived from different individuals, highlighting a limitation that future research needs to address by directly correlating tumor and platelet samples from the same patients. The study highlights the role of tumor-educated platelets (TEPs), which carry RNA signatures reflective of tumor-derived material, providing a non-invasive source for early-detection biomarkers [14,15]. The biomarkers identified from peripheral blood mononuclear cells (PBMCs) showed similar gene expression in tumor tissue samples, emphasizing the role of the identified immunological biomarkers as potential TEPs. TEPs are blood platelets that have absorbed RNA molecules from tumors, allowing them to reflect the gene expression changes occurring within the tumors [16]. This provides a valuable and non-invasive means of detecting cancer at an early stage. Our research enhances the understanding of HNSCC’s genomic architecture and sets the stage for future clinical applications that could significantly improve early diagnosis and treatment outcomes.

## 2. Materials and Methods

### 2.1. Gene Expression Dataset

RNA-seq data for HNSCC was extracted from GEO with accession GSE183635 [17]. The GEO database (RRID:SCR_005012), short for Gene Expression Omnibus database (https://www.ncbi.nlm.nih.gov/geo/query/acc.cgi?acc=GSE183635 (accessed on 7 October 2023)), is a publicly accessible online repository that contains high-throughput gene expression and other functional genomics data. It is maintained by the National Center for Biotechnology Information (NCBI) and is an essential resource for researchers studying gene expression, regulation, and function. This dataset includes RNA-sequencing data for 2351 blood platelet samples of 1628 human patients with stages I–IV cancer spanning 18 different tumor types and 723 asymptomatic and symptomatic controls. Out of the 1628 tumor samples with varying types of cancer, only 101 corresponding to HNSCC (stage I = 4, stage II = 7, stage III = 30, stage IV = 60) and 143 asymptomatic controls were selected for our analysis (Figure S1). Demographic information about the group, age, cancer stage, and sex are provided in Table S1.

### 2.2. Raw Data Preprocessing

Raw sequencing reads were extracted from GEO for extensive preprocessing and data exploration. The preprocessing was performed using the Nextflow [18] nf-core/rnaseq pipeline [19]. The read quality control was performed with FastQC [20], and UMI extraction was performed with UMI tools [21]. Trim Galore (RRID:SCR_011847) [22] was used for adapter and quality trimming, further genome contaminants were removed with BBSplit [23], and ribosomal RNA was removed using SortMeRNA [24]. Alignment and quantification were performed with STAR [25] and salmon [26], respectively, using the GRCh38 genome. Further steps included sorting and indexing alignments with SAMtools (RRID:SCR_002105) [27] and UMI-based deduplicating and duplicate read marking with UMI-tools [21] and Picard MarkDuplicates [28], respectively. Transcript assembly and quantification were performed with StringTie [29]. Finally, BEDTools (RRID:SCR_006646) [30] and bedGraphToBigWig [31] were used to create bigwig coverage files, and extensive quality control of results was performed with RSeQC [32], Qualimap [33], dupRadar [34], Preseq [35], and DESeq2 (RRID:SCR_000154) [36].

### 2.3. Identification of Differentially Expressed Genes and Transcription factors

RStudio (version 4.2) was employed, which provided a robust framework for handling and processing our data. Within this environment, the Bioconductor package DESeq2 (RRID:SCR_006442) was used to identify the differentially expressed genes (DEGs) with the default settings [36]. This approach allowed us to confidently identify genes whose expression levels significantly differed between the cancerous and non-cancerous samples, thereby providing insights into potential biomarkers for head and neck squamous cell carcinoma. A standard volcano plot was constructed to visualize the differential expression of genes by plotting the log2-fold change (±0.5) against the negative log10 of the adjusted *p*-values from RNA-seq data analysis. For the mitochondrial-specific volcano plot, an expanded list of mitochondrial-related genes, categorized by functions such as oxidative phosphorylation, fatty acid oxidation, and the TCA cycle, among other functions, was used [37]. Genes were annotated as mitochondrial-related if they appeared in this list. Upregulated mitochondrial genes were identified by filtering for those with positive log2-fold changes [38]. These genes were highlighted in different colors based on their mitochondrial function categories. The top 10 most significant upregulated mitochondrial genes, determined by the lowest adjusted *p*-values, were labeled on the plot. Furthermore, transcription factors activity inference analysis was conducted using the decoupleR Univariate Linear Model (ULM) with the CollecTRI gene regulatory network for the DEGs [39,40].

### 2.4. Construction of Gene Co-Expression Network

Weighted gene co-expression network analysis (WGCNA) [41] was performed using the R Comprehensive R Archive Network (CRAN) package WGCNA. The soft threshold for network construction was set at 30, and the max block size was set at 25,000. The topological overlap measure was used as signed with a merge cut height of 0.25. Hierarchical clustering was utilized to generate a hierarchical clustering tree of genes representing modules and genes, respectively [42]. After determining the gene module using the dynamic cutting method, the eigenvector value of each module was calculated, and the modules were subsequently analyzed to merge similar modules into new modules. Furthermore, the relationship between constructed network modules and phenotype (disease state and tumor stage) was analyzed. By using large-scale gene expression data to find co-expression networks, hub genes, and potential biomarkers that may be useful for diagnosis, prognosis, or treatment, WGCNA provided a potent method for biomarker identification [43,44].

### 2.5. Enrichment and Pathway Analysis

The analysis of canonical pathways and disease and function associations was performed using Ingenuity Pathway Analysis (IPA) on differentially expressed genes (DEGs) identified by DESeq2, with log2-fold change (logFC) thresholds of +2 and −2 and adjusted *p*-values less than 0.05. Canonical pathway analysis in IPA utilized the z-score algorithm to predict pathway activation or inhibition, and disease and function analysis associated DEGs with known biological functions and diseases, visualized through bar charts and network diagrams. For pathway activity inference, the decoupleR package (version 1.5.0) and the PROGENy model with human-specific weights were used, focusing on the top 500 genes ranked by *p*-value [39]. Pathway enrichment scores were inferred using the Multivariate Linear Model (MLM) method, with t-values indicating pathway activation or inhibition. The JAK-STAT pathway was specifically visualized to confirm activation. Additionally, ligand–receptor interactions were studied using the LIANA package, applying a co-regulation assumption to the bulk RNA dataset [45]. LIANA provided a prior knowledge network, and decoupler inferred significant ligand–receptor interactions by calculating weighted mean expression levels standardized against a background distribution, integrating ligand–receptor interaction knowledge into the analysis.

### 2.6. Machine Learning

A machine learning approach was employed to analyze gene expression data for the classification and biomarker discovery using HNSCC data. The dataset was split into training and testing sets, with 70% of the data used for training and 30% for testing. To ensure the robustness of the model, the genes were filtered based on variance, selecting the top 500 most variable genes for further analysis. The MLSeq package was utilized to implement various classification algorithms, including support vector machines (SVMs) with a radial basis function kernel, nearest shrunken centroids (NSCs), and other classifiers such as PLDA and voom-based methods [46]. For the SVM model, hyperparameter tuning was performed using a grid search approach with 10-fold cross-validation repeated 10 times (repeatedcv). The training process involved applying the variance-stabilizing transformation (VST) from DESeq2 for data preprocessing. The model performance was evaluated on the test set, and key metrics such as accuracy, sensitivity, and specificity were calculated from the confusion matrix. In addition to the SVM model, other classifiers were trained and evaluated using similar cross-validation techniques. The best-performing models were compared based on their accuracy and sparsity. The classifiers included in the comparison were SVM, NSC, PLDA, and voom-based methods (voomDLDA and voomNSC). Feature selection was conducted, and the selected genes from each classifier were visualized using a Venn diagram to identify common biomarkers. 

### 2.7. Network Analysis and Hub Genes Identification

Network analysis allows the identification of the essence of functional relationships shared among genes of interest. This analysis aims to systematically integrate observations from high-throughput experiments for a global understanding of cellular functions under changing environmental conditions. Cytoscape (RRID:SCR_003032) (39) enables the identification of topological parameters of the network using the NetworAnalyzer App (40). We used output from STRING network analysis as the input for further analysis [47]. To identify the potential hub genes, the Cytohubba plug-in (41), employed at default settings, ranked nodes within the network based on features such as degree, edge percolated component, maximal clique centrality (MCC), betweenness centrality, bottleneck centrality, eccentricity, radiality, and stress, thereby helping to identify hub genes.

### 2.8. Gene Signature and isoform Expression Anaysis

Gene expression data for head and neck squamous cell carcinoma (HNSCC) were sourced from The Cancer Genome Atlas (TCGA). We specifically extracted gene expression quantification obtained through STAR-Counts using the TCGAbiolinks package in R. Our analysis focused on a preselected gene signature comprising nine genes: *AGBL5*, *BOP1*, *DDX24*, *FTSJ3*, *GRWD1*, *GTPBP4*, *LGALS1*, *NOP58*, and *RRS1*, identified for their relevance in oncogenic processes in squamous cell carcinoma. Using the TCGAbiolinks and edgeR packages in R, raw counts were normalized to counts per million (CPM) and log-transformed to stabilize variance. The dataset included 44 normal and 406 tumor samples, stratified into stages I through IVC (stage I: 20, II: 98, III: 105, IVA: 265, IVB: 11, IVC: 7). Each tumor stage was compared to the normal group using two-tailed *t*-tests with a Bonferroni correction for multiple comparisons. Significance was reported at various levels, categorized as follows: non-significant (*p* > 0.05), * (*p* ≤ 0.05), ** (*p* ≤ 0.01), and *** (*p* ≤ 0.001). Results were visualized using ggplot2, with boxplots annotated to display sample counts and *p*-values, providing insights into the data’s composition and highlighting significant differences across stages. Further survival analysis and individual gene expression analysis were performed using GEPIA2 [48] and UALCAN [49] on TCGA and GTEx data consisting of 519 HNSCC tumor samples and 44 normal samples. All 563 samples, including the 519 HNSCC samples, from the TCGA and GTEx databases were used for gene signature validation. Individual gene expression patterns at different cancer stages were analyzed using only the TCGA HNSCC dataset (normal = 44, stage I = 27, stage II = 71, stage III = 81, and stage IV = 264). Overall survival analysis was conducted for a combined prioritized gene set with high and low group cutoffs of 75 and 25, respectively. Finally, isoform analysis was performed using GEPIA2 to check the gene expression of specific isoforms of each prioritized gene. GEPIA2 was used to perform the isoform analysis on the selected diagnostic biomarker genes of interest. Isoform analysis was performed on all 9 genes using the HNSCC dataset from the TCGA dataset.

## 3. Results

### 3.1. Transcriptional Landscape of Platelets in Head and Neck Cancer

**Figure 1:**
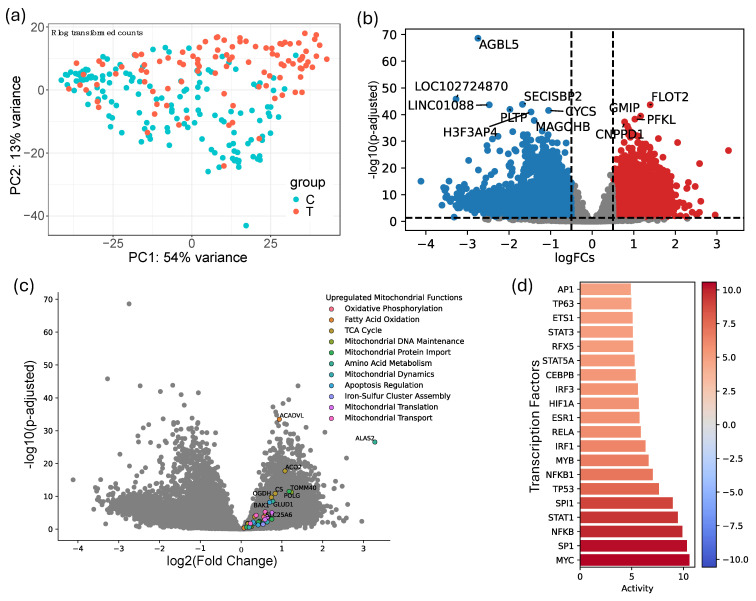
Transcriptional landscape of blood platelets in head and neck cancer patients and healthy controls. (**a**) Principal component analysis (PCA) of rlog-transformed counts showing clear separation between cancer samples (red) and control samples (cyan). (**b**) Volcano plot representing differentially expressed genes between tumor and control samples. (**c**) Volcano plot highlighting genes associated with mitochondrial function in different colors and top upregulated genes, annotated in the figure, that were upregulated in tumor samples. (**d**) Expression levels of key transcription factors involved in cellular proliferation and survival, analyzed in tumor versus control samples.

To understand the influence of head and neck cancer on the transcriptional landscape of blood platelets, RNA-seq data from blood platelets of head and neck cancer patients and healthy controls were analyzed. Initially, principal component analysis (PCA) of the rlog-transformed counts was performed to assess the overall variability in the dataset. The PCA plot demonstrates a clear separation between cancer samples (red) and control samples (cyan), accounting for 54% and 13% of the variance along the first and second principal components, respectively, suggesting distinct transcriptional profiles in platelets from cancer patients versus healthy controls, with no apparent batch effects (Figure 1a).

A volcano plot representing the differentially expressed genes between tumor and control samples highlights the significant changes in gene expression (Figure 1b). Among the most significantly downregulated genes in tumor samples were *AGBL5*, *SECISBP2*, and *PLTP*, which are involved in protein deglutamylation, selenoprotein synthesis, and lipid metabolism, respectively, and their downregulation may impact platelet function and stability in cancer [50,51,52]. Conversely, genes such as *FLOT*, *GMIP*, and *PFKL* showed marked upregulation in tumor samples. FLOT and GMIP are associated with cytoskeletal organization and signal transduction, while *PFKL* plays a key role in glycolysis, suggesting enhanced metabolic and structural adaptability of platelets in the tumor microenvironment [53,54].

Given the critical role of mitochondria in cellular energy metabolism and the known metabolic alterations in cancer, we further investigated the mitochondrial function of platelets in this context [55]. Analysis revealed an upregulation of genes associated with oxidative phosphorylation, fatty acid oxidation, and mitochondrial dynamics (Figure 1c). The increased expression of these mitochondrial-related genes indicates potential metabolic reprogramming in platelets induced by the tumor environment, likely essential for meeting the altered metabolic demands of cancer and supporting tumor progression.

In addition to mitochondrial function genes, several transcription factors related to inflammation and cancer were highly upregulated in tumor samples (Figure 1d). Notably, MYC, SP1, and NFKB, key regulators of cell proliferation, survival, and immune response, showed significant upregulation [56,57]. The enhanced activity of these transcription factors suggests their pivotal role in modulating platelet function in the context of cancer, potentially contributing to the tumor-supportive phenotype observed in cancer-associated platelets. The observed transcriptional changes in platelets from head and neck cancer patients indicate a systemic impact of the tumor on the blood microenvironment.

### 3.2. Pathwnalysis and Functional Implications in Tumor-Educated Platelets

**Figure 2:**
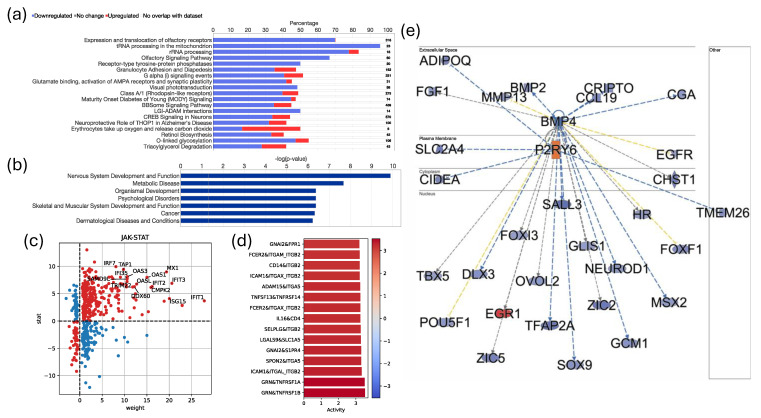
Pathway and functional analysis of differentially expressed genes in blood platelets from head and neck cancer patients. (**a**) Top enriched canonical pathways identified using IPA. The *x*-axis represents the percentage of differentially expressed genes (DEGs) in tumors versus healthy samples, with blue bars indicating downregulated genes and red bars indicating upregulated genes. (**b**) Disease and function analysis showing significant association of DEGs with cancer and metabolic diseases, suggesting tumor-induced transcriptional changes in platelets. (**c**) Significant upregulation of the JAK-STAT pathway and its associated genes in tumor samples, indicating roles in cell proliferation, survival, and immune response. (**d**) Receptor–ligand signaling analysis revealing upregulation of GRN-TNFRSF1b and ICAM1-ITGAL_ITGB2 signaling, known for roles in inflammation and cellular adhesion. (**e**) Network analysis of *BMO4* and *P2RY6* genes showing downregulation of most genes, except for the upregulation of EGR1 and P2RY6, which are involved in growth, differentiation, immune, and inflammatory responses.

We further analyzed the pathways altered in the platelets of tumor samples compared to normal samples using the commercial IPA analysis toolkit (Figure 2a). The *x*-axis represents the percentage of differentially expressed genes (DEGs) in tumors versus healthy samples. Blue bars indicate downregulated genes, and red bars represent upregulated genes. Notably, pathways involved in olfactory receptor expression and translocation, rRNA processing, olfactory signaling, and granulocyte adhesion and diapedesis were significantly downregulated, suggesting functional suppression in the platelets of cancer patients. Disease and function analysis revealed that DEGs were significantly associated with cancer and metabolic diseases, indicating that the tumor influences platelets, altering their transcriptional landscape to resemble cancer cells (Figure 2b). This supports the concept of tumor-educated platelets (TEPs), where platelets are altered by the tumor microenvironment to aid in cancer progression.

Subsequent analysis showed significant upregulation of the JAK-STAT pathway and its associated genes in tumor samples, crucial for cell proliferation, survival, and immune response, suggesting that platelet activation may support tumor growth and immune evasion (Figure 2c) [58]. Additionally, receptor–ligand signaling analysis revealed upregulation of GRN-TNFRSF1b and ICAM1-ITGAL_ITGB2 signaling in platelets, known to play roles in inflammation and cellular adhesion (Figure 2d) [59,60]. Network analysis of BMO4 and P2RY6 genes showed that most genes were downregulated except for EGR1 and P2RY6, which were upregulated (Figure 2e). *EGR1*, a transcription factor involved in growth and differentiation, and *P2RY6*, a receptor gene involved in immune and inflammatory responses, indicate significant transcriptional changes in platelets potentially aiding tumor manipulation of the host environment [61,62]. These findings highlight extensive transcriptional reprogramming of platelets in head and neck cancer, encompassing metabolic pathways, immune response, and cellular signaling.

### 3.3. WGCA and Machine Learning Approach to Identify Diagnostic Biomarker Targets

**Figure 3:**
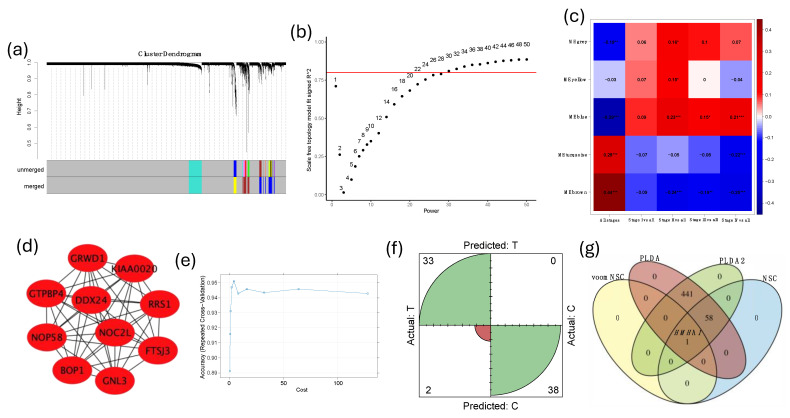
Identification of diagnostic biomarker targets using WGCA and machine learning. (**a**) Clustering analysis using the average linkage method and Pearson correlation to assess relationships and group similarities among all samples. (**b**) Network construction with a soft-thresholding power of 30. (**c**) Heatmap representing the correlation of cancer stage with different modules. (**d**) Identification of top ten hub genes (*FTSJ3*, *BOP1*, *DDX24*, *GRWD1*, *RRS1*, *NOC2L*, *KIAA0020*, *NOP58*, *GTPBP4*, and *GNL3*) using the STRING database network and Cytohubba plug-in in Cytoscape. (**e**) Performance of the SVM Radial classifier, achieving an accuracy of 98.02% with 95% confidence interval and a sensitivity of 94.3% after hyperparameter tuning using a grid search approach with 10-fold cross-validation repeated 10 times. (**f**) Confusion matrix evaluating false positive and negative predictions. (**g**) Venn diagram comparing four machine learning algorithms, identifying common genes used for prediction, with *AGBL5* and *HMHA1* being the top genes and *HMHA1* being utilized by all models for classification, highlighting its potential role in platelets. Statistical significance is indicated as follows: * *p* < 0.05, ** *p* < 0.01, *** *p* < 0.001.

To identify diagnostic biomarker targets, we employed a weighted gene co-expression network analysis (WGCNA) and machine learning approach. Clustering analysis using the average linkage method and Pearson correlation was conducted to assess relationships and group similarities among all samples (Figure 3a). A network was constructed with a soft-thresholding power of 30 (Figure 3b). Among the various modules identified, the brown module was selected for detailed examination due to its strong association with both tumor stage and grade across stages I to IV, with statistically significant *p*-values (Figure 3c). This suggests that the brown module contains genes pivotal in early tumor development and progression, making them promising candidates for early diagnostic biomarkers.

Further analysis integrated the STRING database network and Cytohubba plug-in in Cytoscape, using the maximal clique centrality (MCC) method to identify pivotal hub genes within the head and neck cancer molecular interactions. The top ten hub genes identified were *FTSJ3*, *BOP1*, *DDX24*, *GRWD1*, *RRS1*, *NOC2L*, *KIAA0020*, *NOP58*, *GTPBP4*, and *GNL3* (Figure 3d). These genes are critical nodes within the network, reflecting their substantial influence on cellular processes linked to cancer progression, and marking them as potential targets for therapeutic intervention or early-detection biomarkers.

To differentiate between tumor and normal tissue samples, we implemented the ML-Seq framework with various machine learning classifiers. Among the six classifiers assessed—voomNSC, PLDA, PLDA2, NSC, SVM Radial, and NBLDA—the SVM Radial classifier emerged as the best performer. It achieved an accuracy of 98.02% with a 95% confidence interval and a sensitivity of 94.3% after hyperparameter tuning using a grid search approach with 10-fold cross-validation repeated 10 times (Figure 3e). The confusion matrix was plotted to evaluate false positive and negative predictions (Figure 3f).

A comparison of four machine learning algorithms and a Venn diagram of common genes used for prediction revealed that *AGBL5* and *HMHA1* were the top genes used by most models, with *HMHA1* being the only gene utilized by all models for classification, highlighting its potential role in platelets (Figure 3g). These metrics underscore the classifier’s robustness in distinguishing tumors from normal samples, emphasizing its potential for clinical diagnostic applications.

### 3.4. Evaluation of Diagnostic Biomarker Genes in HNSCC Tumor Samples

**Figure 4:**
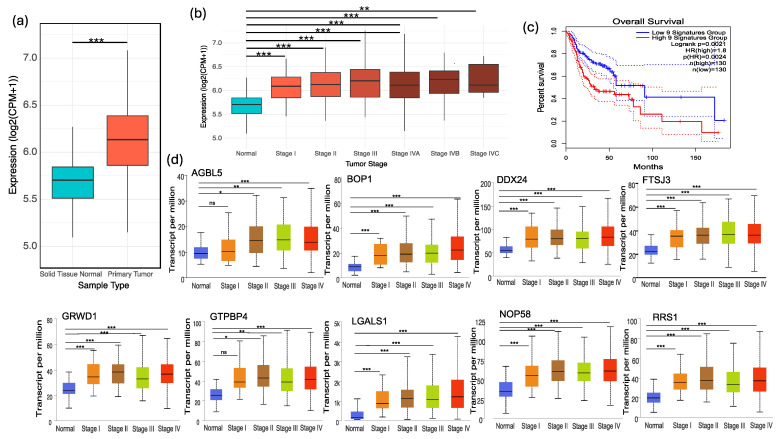
Assessment of diagnostic biomarker genes in HNSCC tumor samples. (**a**) Expression levels of the gene signature in HNSCC samples compared to normal tissue samples from TCGA, demonstrating significantly elevated expression in HNSCC samples. (**b**) Expression of the gene signature across HNSCC stages, showing consistent upregulation in tumor samples. (**c**) Survival analysis of the diagnostic biomarker gene signature, indicating a significant correlation between gene expression levels and patient survival rates, with lower expression levels associated with better prognosis. (**d**) Stage-wise comparative analysis of gene expression within the biomarker set across the four stages of HNSCC progression, showing consistent expression levels across all stages with some genes exhibiting increased expression correlating with advancing cancer stages. Statistical significance is indicated as follows: * *p* < 0.05, ** *p* < 0.01, *** *p* < 0.001.

We rigorously assessed the proposed diagnostic biomarker genes using HNSCC tumor samples from The Cancer Genome Atlas (TCGA) and the Genotype-Tissue Expression (GTEx) project, addressing the scarcity of blood-based RNA-seq data. Our analysis revealed that the expression levels of the gene signature were significantly elevated in HNSCC samples when compared to the normal tissue samples, demonstrating the genes’ diagnostic robustness (Figure 4a). Further dissection of the gene signature expression across HNSCC subtypes illustrated consistent upregulation in tumor samples, reinforcing the signature’s broad applicability as a diagnostic tool (Figure 4b). This consistency across various HNSCC subtypes underscores the signature’s potential utility as a universal marker of tumor presence, regardless of the tumor’s specific subtype.

Survival analysis was conducted on the diagnostic biomarker gene signature to assess its prognostic value and impact on patient outcomes (Figure 4c). A statistically significant correlation was found between the expression levels of these genes and patient survival rates. Notably, patients exhibiting lower expression levels of the biomarker genes had a markedly better prognosis and extended survival times than those with higher expression levels. This association highlights the potential clinical significance of the gene signature, indicating its capacity to function as a diagnostic tool and its utility in prognostication. The relationship between gene expression and patient survival underscores the possibility that these genes could be integral to the pathophysiology of the disease. It points to a dual role of these biomarkers: as indicators for early detection and as predictors of disease trajectory. While promising, these findings necessitate further investigation to understand the genes’ roles in head and neck cancer progression, potentially illuminating new therapeutic pathways and guiding treatment decisions for HNSCC.

Furthermore, we conducted a stage-wise comparative analysis for each gene within the biomarker set, charting gene expression across the four stages of HNSCC progression (Figure 4d). While the genes maintained relatively consistent expression levels across all stages, indicative of their early diagnostic potential, some genes demonstrated a trend of increased expression correlating with advancing cancer stages.

### 3.5. Isoform Analysis for Diagnostic Biomarker Genes in HNSCC

**Figure 5:**
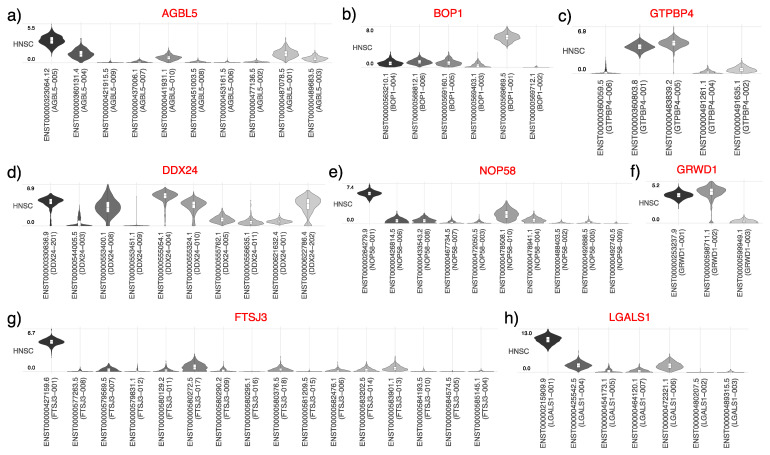
Isoform expression of selected genes in HNSCC. (**a**–**h**) The violin plot shows the expression levels of different isoforms of each gene in HNSCC samples. Each plot represents the average expression level of a specific isoform across HNSCC samples.

Isoform-specific expression patterns of the nine genes identified as diagnostic biomarkers for HNSCC were meticulously analyzed and visualized, with cancer samples plotted along the *x*-axis and isoform expression along the *y*-axis (Figure 5a–h). This isoform-level scrutiny allowed for pinpointing the most prevalently expressed isoforms within the HNSCC patient cohort, thereby revealing potential targets for therapeutic intervention. 

The analysis revealed a distinct overexpression of a single isoform for several genes, namely *AGBL5*, *BOP1*, *FTSJ3*, *LGALS1*, and *NOP58*, suggesting a dominant role of these isoforms in the cancer phenotype. The observed isoform consistency underscores their stability across different cancer types and discounts the likelihood of isoform switching events in HNSCC, which could indicate a cancer-specific expression pattern and be potentially relevant for targeted therapies. Conversely, the *DDX24* gene presented a more complex expression profile, with two out of five isoforms markedly overexpressed, indicating a potentially significant role for these isoforms in HNSCC pathology. Similarly, the *GRWD1* gene showed overexpression in two of its three isoforms. This pattern was mirrored in the GTPBP4 gene, where two out of five isoforms were predominantly expressed in the HNSCC samples.

The consistency of isoform expression, coupled with the specific overexpression of certain isoforms, may contribute to the oncogenic process and serve as a hallmark of the disease. These findings illustrate a nuanced expression landscape, where identifying dominant isoforms could guide the development of isoform-specific diagnostic and therapeutic strategies. The gene-specific isoform expression and association with HNSCC could reflect critical alterations in gene regulation mechanisms pivotal to cancer development and progression. 

## 4. Discussion

Despite considerable progress in therapeutic modalities, cancer continues to be marked by high mortality, primarily due to its heterogeneity [63]. Even as surgery remains a mainstay treatment, the efficacy of alternative therapies like radiation, chemotherapy, gene therapy, and targeted therapy heavily depends on the precise identification of therapeutic targets [64]. Transcriptional analyses in head and neck cancer (HNC) shed light on its molecular intricacies, offering new vistas in disease management. Yet, understanding specific molecular pathways active in HNC remains elusive. Dissecting these pathways is vital for pinpointing target regulators and their roles in sustaining oncogenic processes and navigating the complexities of cellular death, survival, and cancer-promoting mechanisms.

Network biology approaches are increasingly applied across various diseases to identify biomarkers by exploring complex molecular interactions. Our study harnessed systems and network biology to understand the intricate genome-level landscapes that could inform targeted treatment strategies in HNC. RNA-seq analysis delineated gene expression variances in control versus disease states. Through WGCNA, we identified genes in modules notably associated with cancer status, specifically pinpointing the brown module as significantly correlated with cancer. Network analysis further narrowed down nine hub genes: *DDX24*, *GTPBP4*, *BOP1*, *NOP58*, *GRWD1*, *RRS1*, *KIAA0020*, *GNL3*, and *FTSJ3*.

The identified hub genes exhibit multifaceted roles in oncogenesis. *DDX24*, *GTPBP4*, and *BOP1*, for example, are involved in RNA metabolism, cell-cycle regulation, and ribosome biogenesis, respectively, reflecting their substantial influence on cellular processes linked to cancer progression [65,66,67,68]. These genes are not only potential targets for therapeutic intervention but also promising candidates for early diagnostic biomarkers due to their significant association with cancer status.

Our results demonstrate that the proposed candidate biomarkers exhibit similar expression patterns in both blood platelets and tumor tissues, suggesting a sophisticated mechanism through which tumors can influence and ’educate’ circulating platelets [14,15,69]. This education process results in platelets that carry biomarkers reflective of the tumor’s transcriptomic and epigenetic landscape and may actively participate in cancer progression. The significant overlap in gene expression underscores the bidirectional communication between tumor cells and platelets via exosomes and microRNAs, facilitating the transfer of tumor-derived signals to the platelets [70]. Such interactions enable platelets to mirror the tumor environment, presenting a less invasive source for crucial diagnostic markers. This insight supports the potential of leveraging tumor-educated platelets (TEPs) in liquid biopsy approaches, providing a window into tumor behavior through blood samples.

Additionally, our pathway analysis revealed the extensive transcriptional reprogramming of platelets in head and neck cancer. Notably, pathways involved in olfactory receptor expression, rRNA processing, and granulocyte adhesion and diapedesis were downregulated, while the JAK-STAT pathway and genes related to mitochondrial function were upregulated. The downregulation of olfactory pathways and granulocyte adhesion suggests functional suppression in platelets, likely as an adaptive response to the tumor microenvironment [71]. In contrast, the upregulation of the JAK-STAT pathway and mitochondrial function genes indicates an enhanced metabolic and proliferative state, supporting the tumor’s growth and immune evasion strategies [72,73].

Our survival analysis of the diagnostic biomarker gene signature showed a statistically significant correlation between gene expression levels and patient survival rates. Patients with lower expression levels of the biomarker genes had a markedly better prognosis and extended survival times compared to those with higher expression levels. Furthermore, our stage-wise comparative analysis of gene expression within the biomarker set across the four stages of HNSCC progression revealed that while the genes maintained relatively consistent expression levels across all stages, some demonstrated increased expression correlating with advancing cancer stages. This observation hints at their additional role in tracking disease progression and could be a focus for future prognostic studies.

Early detection of HNSCC, which currently lacks precise and early diagnostic biomarkers, can profoundly impact patient outcomes by enabling earlier and more targeted therapeutic interventions. The gene signatures identified could be developed into practical diagnostic tests that may be less invasive than current methods, providing a critical advantage in clinical settings. Our findings contribute to a growing body of evidence that seeks to demystify the complex molecular underpinnings of HNC. The clinical translation of these findings could advance the approach to patient management in HNC, paving the way for more personalized and efficacious treatments. However, a limitation of this study is that platelet samples and tumor samples were derived from different individuals, highlighting the need for future research to directly correlate tumor and platelet samples from the same patients. This will further elucidate the relationship between tumors and platelets, advancing our understanding of tumor-educated platelets. Further wet lab experiments and validation are required to fully understand the functions of the signature genes and their potential use as biomarkers and/or therapeutic targets.

## 5. Conclusions

In conclusion, our integrative study using systems and network biology, alongside machine learning approaches, has identified key biomarkers and pathways involved in head and neck cancer (HNC). We pinpointed ten critical hub genes—*DDX24*, *GTPBP4*, *BOP1*, *NOP58*, *GRWD1*, *NOC2L*, *RRS1*, *KIAA0020*, *GNL3*, and *FTSJ3*—that show significant potential as diagnostic biomarkers. These genes exhibit differential expression patterns in cancerous versus healthy tissues, highlighting their importance in early detection and targeted therapy. Additionally, our findings reveal the extensive transcriptional reprogramming of platelets in HNC patients, suggesting that tumor-educated platelets (TEPs) can mirror the tumor’s transcriptomic and epigenetic landscape. This highlights the potential of TEPs in liquid biopsy approaches, offering a minimally invasive method for cancer diagnostics. Our pathway analysis demonstrated significant alterations in key biological pathways, such as the JAK-STAT pathway and mitochondrial function genes, supporting the tumor’s metabolic and proliferative needs. While our study uses platelet and tumor samples from different individuals, future research should aim to directly correlate these samples from the same patients to deepen our understanding of TEPs. These findings pave the way for developing precise diagnostic tests and personalized treatment strategies, ultimately aiming to improve patient outcomes and quality of life. Further experimental validation is required to fully elucidate the roles of these biomarkers in HNC and to confirm their clinical utility.

## Data Availability

The datasets GSE183635 for this study can be found in the GEO repository under accession GSE183635 [https://www.ncbi.nlm.nih.gov/geo/query/acc.cgi?acc=GSE183635 (accessed on 22 June 2024)].

## Supplementary Materials

The following supporting information can be downloaded at: https://www.mdpi.com/article/10.3390/cancers16132399/s1, Figure S1: Comprehensive workflow of bioinformatics analysis for identifying and validating diagnostic characteristic genes; Table S1: Demographic and clinical characteristics of HNSCC samples used in the study.
