# Supplementary figures and images for: Immunological Signatures for Early Detection of Human Head and Neck Squamous Cell Carcinoma through RNA Transcriptome Analysis of Blood Platelets
Source: Cancers (Basel). 2024 Jun 29;16(13):2399. doi: 10.3390/cancers16132399 (PMC11240534; doi:10.3390/cancers16132399)

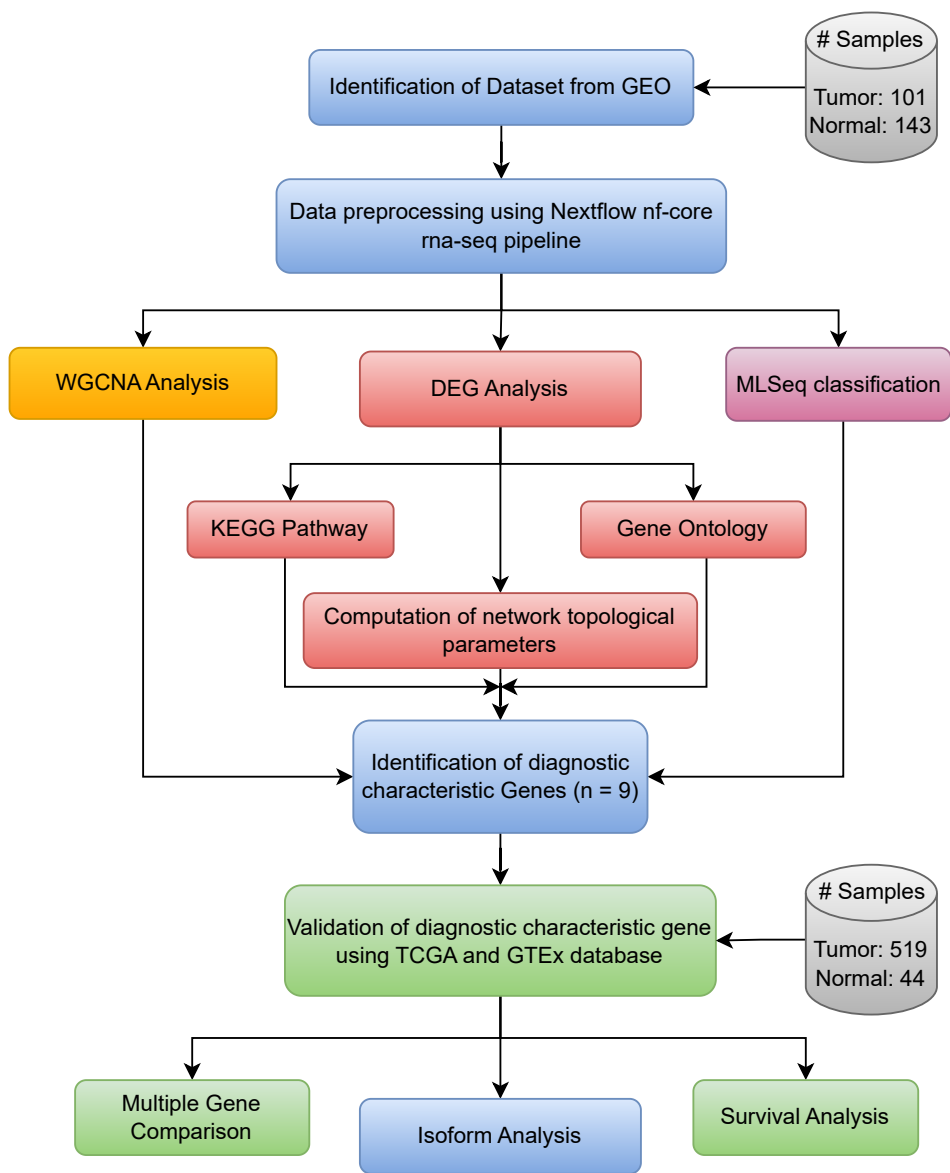

Supplement: Supplementary file 1 [file cancers-16-02399-s001.zip › Figure S1.pdf]
